# An O-Specific Polysaccharide/Tetanus Toxoid Conjugate Vaccine Induces Protection in Guinea Pigs against Virulent Challenge with *Coxiella burnetii*

**DOI:** 10.3390/vaccines10091393

**Published:** 2022-08-25

**Authors:** Stephen R. Graves, Aminul Islam, Lawrence D. Webb, Ian Marsh, Karren Plain, Mark Westman, Xavier A. Conlan, Rodney Carbis, Rudolf Toman, John Stenos

**Affiliations:** 1 Australian Rickettsial Reference Laboratory, University Hospital, Geelong, VIC 3220, Australia; 2 Elizabeth Macarthur Agricultural Institute, NSW Department of Primary Industries, Menangle, NSW 2567, Australia; 3 School of Life and Environmental Science, Deakin University, Waurn Ponds, VIC 3216, Australia; 4 Symbioticus Pty Ltd., Strathmore, VIC 3041, Australia; 5 Institute of Virology, Slovak Academy of Sciences, 845 05 Bratislava, Slovakia

**Keywords:** conjugate vaccine, Q-fever, *Coxiella burnetii*, axenic media

## Abstract

Q fever is caused by the bacterium *Coxiella burnetii* and is spread to humans from infected animals especially goats, sheep and cattle, predominantly when giving birth. There is an effective human vaccine (Q-VAX) against Q fever, and although Q fever is a worldwide problem, the vaccine is only used in Australia due to difficulties associated with its use and the risk of adverse reactions. The desire to protect humans, particularly farmers and abattoir workers, from Q fever prompted the development of a new safe and effective human vaccine without all the difficulties associated with the current vaccine. Candidate vaccines were prepared using purified O-specific polysaccharide (OSP) extracted from the lipopolysaccharide of virulent (phase 1) *C. burnetii*, strain Nine Mile, which was then conjugated to a tetanus toxoid (TT) carrier protein. Two vaccines were prepared using OSP from *C. burnetii* grown in embryonated eggs (vaccine A) and axenic media (vaccine B). Vaccines with or without alum adjuvant were used to vaccinate guinea pigs, which were later challenged by intranasal inoculation with virulent *C. burnetii*. Both vaccines protected guinea pigs from fever and loss of weight post challenge. Post-mortem samples of the spleen, liver and kidney of vaccinated guinea pigs contained substantially less *C. burnetii* DNA as measured by PCR than those of the unvaccinated control animals. This study demonstrated that a *C. burnetii* OSP-TT conjugate vaccine is capable of inducing protection against virulent *C. burnetii* in guinea pigs. Additionally, OSP derived from *C. burnetii* grown in axenic media compared to OSP from embryonated eggs is equivalent in terms of providing a protective immune response.

## 1. Introduction

Q fever was recognised as a distinct, zoonotic, infectious disease of humans last century, with it being first recognised in Australia in 1937 [1] in abattoir workers exposed to pregnant cattle. The causative agent *Coxiella burnetii* was first isolated from a tick in the USA [2] and a later human isolate [3] was shown to be identical. *C. burnetii* infects many species of vertebrate animals, causing coxiellosis, although the clinical and economic significance is often not great. The main problem occurs when humans become infected. The latter infection is generally self-limiting and is characterised by an acute onset of fever, myalgia, headache and severe fatigue. However, in a small number of cases infection can become chronic, causing debilitating symptoms and in severe cases, infection can result in death [4].

Q fever is predominantly a disease of rural populations due to the greater exposure of humans to infected animals [5] and the presence of *C. burnetii* circulating in the environment [6]. *C. burnetii* is geographically widely distributed and is present in most countries. Reports of Q fever incidence are considered underestimates because the infection is very difficult to diagnose and most patients spontaneously recover after 2–3 weeks [7], often resulting in the search for a definitive diagnosis being abandoned. 

The lipopolysaccharide (LPS), or more specifically the O-specific polysaccharide (OSP), of virulent phase I *C. burnetii* consists of an inner core oligosaccharide with additional terminal sugars, include two unique sugars, virenose and dihydrohydroxystreptose, which are believed to be critical in the development of a protective immune response. When extensively passaged, *C. burnetii* can undergo a phase change from virulent phase I to avirulent phase II, characterised by a severe truncation of its LPS where only the lipid A and a small part of the core oligosaccharide remains. Phase II bacteria would not be suitable for vaccine use, so care must be taken to ensure that seed bacteria and production cultures consist of phase I *C. burnetii*.

There were several attempts at producing a human vaccine against Q fever. Efforts in Europe [8,9,10] and the USA [11,12,13,14,15,16,17,18] yielded vaccines that were protective but were unacceptably reactogenic, causing adverse reactions in recipients. Of particular concern were the reactions seen in people receiving a booster dose and in people with prior natural exposure to *C. burnetii*. A formaldehyde inactivated whole-cell vaccine (Q-VAX), made from the phase I Henzerling strain of *C. burnetii*, was manufactured and used in Australia from 1989 [19]. It is an effective vaccine and gives recipients good protection from Q fever but can only ever be given once in a person’s lifetime [20]. Administration of the vaccine is difficult; patients must be screened one week prior to vaccination for pre-existing humoral immunity (serological test) and for cell-mediated immunity (skin test). Any evidence of prior exposure to the *C. burnetii* by either test renders the patient ineligible to receive the vaccine due to the significant risk of an adverse event following vaccination [21]. Due to these requirements, many doctors are reluctant to recommend vaccination against Q fever, even though it still represents a significant infection problem [22,23].

As a consequence of the difficulties in the administration of Q-VAX and the risk of reactogenicity, it was decided to develop a new generation Q fever vaccine. The phase 1 LPS, a component of the bacterial cell wall and recognised virulence factor of *C. burnetii*, was chosen as the target antigen for vaccine development. Intact LPS is, however, an endotoxin and likely to be reactogenic due to its lipid A component. Lipid A was therefore chemically removed, and the remaining O-specific polysaccharide (OSP) was used as the protective antigen. The purified OSP was then conjugated to a tetanus toxoid (TT), with the aim of inducing a T-cell dependent immune response to the OSP. Without conjugation, the OSP would induce a T-cell independent response and would likely be less immunogenic. 

*C. burnetii* is an intracellular bacterium which has been difficult to culture, and Q-VAX is derived from bacteria grown in embryonated hens’ eggs. This type of culture system is problematic if used in vaccine production due to the need for a specialised facility to handle eggs. In this study, we tested vaccines created from OSP derived from *C. burnetii* grown in axenic culture media, and OSP grown in embryonated eggs. Guinea pigs were selected to test the vaccines as infection mimics Q fever in humans with respect to symptoms, duration of illness and recovery [24,25,26].

The vaccines were given intramuscularly (vaccine A) or subcutaneously (vaccine B with an alum adjuvant) to guinea pigs which were later challenged with virulent *C. burnetii* to determine if the vaccines induced protection from infection. In this report, we define the method used to prepare a *C. burnetii* OSP-conjugate vaccine and demonstrate its ability to protect guinea pigs against challenge with virulent bacteria. 

## 2. Materials and Methods

### 2.1. Preparation of Coxiella burnetii Seed

The *C. burnetii* Nine Mile strain was supplied by Dr Nathan Unsworth, Defence Science and Technology Group, Australian Government. Eight wild-type male adult mice (Piper’s Farm, Cowra, NSW, Australia) were injected intraperitoneally with 0.1 mL being approximately 10^4^ *C. burnetii* bacteria. Two mice were euthanised by cervical dislocation on days 7, 9, 11 and 14 after infection. Their enlarged spleens were removed by sterile dissection, and each spleen was individually homogenised by hand in a sterile glass homogeniser (Kimble, Rockwood, TN, USA, Cat # 749610-0015, size 15) in 5mL of sterile Hanks Balanced Salt Solution (HBSS) at room temperature (21 °C). Each infected spleen preparation was tested by quantitative (q)PCR to determine the concentration of *C. burnetii* as genome equivalents. The spleen suspensions were stored at 4 °C.

### 2.2. Growth of C. burnetii in Embryonated Eggs and Preparation of OSP for Vaccine A

Specific pathogen-free eggs were inoculated with phase I *C. burnetii*, and the bacteria were harvested and purified from the chicken embryo membranes as previously described [27]. *C. burnetii* cells (1.5 g wet weight) were suspended in 50 mM Tris-HCl buffer (150 mL, pH 7.5) and treated simultaneously with RNase (EC 3.1.27.5) and DNase (EC 3.1.27.1), both from bovine pancreas (Boehringer, Ingelheim, Germany) at 37 °C for 16 h. The cells were then treated with trypsin 1:250 (EC 232-650-8; Sigma) at 37 °C for 90 min, followed by proteinase K from *Tritirachium album* (EC 3.4.21.14; Sigma, St. Louis, MO, USA) at 37 °C for 16 h. After enzyme treatments, the cell suspension was centrifuged at 14,000× *g*, 10 °C for 50 min and the sediment was washed with acetone. Cells were extracted with chloroform–methanol (2:1, *v*/*v*) at 20 °C for 3 h to remove phospholipids. The extraction was repeated with the fresh solvent mixture for 2 h. The cell suspension was centrifuged at 3000× *g*, 20 °C for 20 min, and the sediment was suspended in preheated distilled water (150 mL, 68 °C) and extracted with an equal volume of aqueous 90% phenol [28]. Lipopolysaccharide (LPS) was obtained from the aqueous phase after extensive dialysis (Serva MWCO 3500) and lyophilisation. Yield of the crude LPS was 144 mg (9.6%), calculated on the weight of the original cells. LPS was further purified by treatments with RNase, DNase and proteinase K (see above), and was dialyzed and lyophilised. 

The protein and nucleic acid content of the purified LPS was less than 1%. Protein content was estimated colorimetrically [29] and nucleic acids were determined spectrophotometrically. Phosphate, 3-deoxy-D-manno-oct-2-ulosonic acid (KDO) and hexosamine contents of the LPS were 140.2, 98.6, and 288.5 nmol/mg, respectively. These were estimated colorimetrically for phosphate [30], KDO [31] and hexosamine [32]. Analysis of the constituent neutral sugars as the corresponding alditol acetates [33] by gas chromatography–mass spectrometry (GC-MS) revealed the presence of virenose (6-deoxy-3-C-methyl-D-gulopyranose), dihydrohydroxystreptose [3-C-(hydroxymethyl)-L-lyxofuranose] [34] D-mannose, D-glucose and D-glycero-D-manno-heptose in 24.2, 14.5, 35.2, 1.5 and 24.6 (mole%), respectively.

LPS (120 mg) was hydrolysed with aqueous 1% acetic acid (100 mL) at 100 °C for 90 min, and the hydrolysate was kept at −20 °C overnight. After melting, the precipitated lipid A was removed by low-speed centrifugation (9300× *g* for 10 min). A solution of delipidated polysaccharide (OPS) was neutralised, extensively dialysed (Serva MWCO 3500) and lyophilised. Yield of OPS was 78.2 mg (65.2%). Phosphate, KDO and hexosamine contents of the OPS were 70.3, 45.2, and 312.6 nmol/mg, respectively. The protein and nucleic acid content of OPS was less than 1%. Analysis of neutral sugars by GC-MS was performed on an SP-2330 (30 m × 0.25 mm; Supelco, Bellefonte, PA, USA) fused silica capillary column and gave virenose, dihydrohydroxystreptose, D-mannose, D-glucose and D-glycero-D-manno-heptose in 21.1, 11.2, 37.3, 3.2 and 27.2 (mole%), respectively.

### 2.3. Growth of C. burnetii in Liquid Axenic Medium and Preparation of OSP for Vaccine B

Liquid ACCM-D medium (Cytiva, Marlborough, MA, USA, cat # RR17784.01) was prepared according to published methods [35]. A starter culture of 500 mL of *C. burnetii* was grown microaerophilically to a concentration of approximately 10^5^/mL. This culture became the 5% inoculum used to inoculate 10 L of ACCM-D medium in a 20 L Wave culture bag installed in a biofermenter (Cytiva, “Wave 25”, USA). *C. burnetii* cells were grown for 14 days at 37 °C, 2.5% oxygen and 5% carbon dioxide with continuous rocking. At the end of bacterial growth, the *C. burnetii* concentration was approximately 10^8^/mL, as determined by qPCR.

*C. burnetii* cells were inactivated by adding formaldehyde to a final volume of 1% and shaking the culture for 3 days. Inactivation was confirmed by three serial passages in VERO cell monolayers, each incubated for 2 weeks, and each showing no cytopathogenic effect (CPE), as well as no increase in *C. burnetii* numbers as demonstrated by weekly qPCR. The *C. burnetii* cells were centrifuged at 15,000× *g* for 30 min and the cell sediment was stored at 4 °C.

Isolation of OSP from *C. burnetii* was performed using published methods [36]. Briefly the cell sediment (1 mg/mL wet weight) was subjected to a mild acid hydrolysis (1% acetic acid, 100 °C for 150 min), then cooled to room temperature and centrifuged at 14,000× *g* for 1 h. The supernatant was separated from the pellet and the pH was increased to 7.0 by addition of 1M sodium hydroxide (NaOH). The supernatant was then heated to 30 °C and sodium deoxycholate added to a final concentration of 0.2%. The mixture was stirred for 2 min before the pH was reduced to 2.0 with 33% HCL. The mixture was held in a water bath at 30 °C for 15 min and the precipitate was removed using a 0.20 µm filter (Corning, Corning, NY, USA, cat # 431218). The solution was then dialysed (Sartorius MWCO 5000) against distilled water then sterilised by filtration through a 0.20 μm filter.

### 2.4. Conjugation of OSP to TT

The purified OSP preparations A and B were firstly activated and then derivatised using adipic acid dihydrazide (ADH) as follows: The pH of the OSP was adjusted to between 5.5 and 6.0, and CDAP (1-cyano-4-dimethylaminopyridinium tetrafluoroborate) (100 mg/mL in acetonitrile) was added to the OSP in a ratio of 1:1 CDAP to OSP to give a final concentration of OSP of 50 μg/mL. The pH of the solution was maintained at between 5.5 and 6.0 for 5 min, then increased to 8.0 by slowly adding sodium hydroxide and holding for 2 min. 

A solution of ADH (90 mg/mL in 0.1 M NaHCO_3_) was then added to the activated OSP with vigorous mixing at a ratio of 4.5mg of ADH for every 1 mg of CDAP/OSP. The mixture was incubated at room temperature for 2 h, maintaining the pH at 8.0. After 2 h, unreacted CDAP and ADH were removed by dialysis (Sartorius MWCO 5000) against two changes of MES (2-(N-morpholino)ethanesulfonic acid) buffer (80 mM, pH 5.6).

Tetanus toxoid (TT) (BioFarma, Indonesia) was conjugated to the derivatised OSP as follows: TT (in 80 mM MES buffer) was added to the derivatised OSP and mixed, and 1-ethyl-3-(-3-dimethylaminopropyl) carbodiimide (EDC) was added so that the final concentrations of TT:OSP:EDC were 17.5:35:140 mg/mL, respectively. The conjugation reaction was 2 h at room temperature, with the pH maintained between 5.5 and 5.8, and the conjugated product was dialysed (Sartorius MWCO 100kD) against three changes of phosphate buffered saline (PBS) to remove any unconjugated polysaccharide. 

### 2.5. Chemical and Physical Analysis of Conjugates

OSP and conjugates were assayed for sugar content using the anthrone assay [37], ADH concentration by TNBS assay [38], protein content by Bradford assay [39] and nucleic acid by ultraviolet spectroscopy. Molecular size was assessed by size exclusion chromatography using Sephacryl S-1000 (GE Healthcare, Marlborough, MA, USA) and an isocratic mobile phase of PBS (ph 7.2). Detection was performed at 280 nm.

### 2.6. Vaccination of Guinea Pigs and Challenge with Virulent C. burnetii

The Elizabeth Macarthur Agricultural Institute animal ethics committee approval (M19/06) was obtained for the use of mice and guinea pigs, which were housed and handled as per their requirements. Temperature microchips (BMDS, IPTT-300, Yuasa Bio Systems Co Ltd., Okayama City, Japan) were inserted subcutaneously into the guinea pigs’ backs. Temperatures were read using a reading wand (BMDS Reader model DAS-8027-IUS).

In the first study (vaccine A), seven male guinea pigs were injected intramuscularly with 30 μg (OSP content) of vaccine A without adjuvant. An unvaccinated control group of four guinea pigs and a second unvaccinated control group of eight animals were included. Three different breeds of guinea pigs were used: American, Silver Agouti and IMVS Coloured, depending on local availability. On day 57 post vaccination, the vaccinated group and the second group of eight unvaccinated guinea pigs were challenged by placing 0.2 mL of homogenised mouse spleen containing 10^6^ to 10^7^ *C. burnetii* cells into the nostrils of lightly anaesthetised animals. Animals were anaesthetised using a mixture of ketamine (10 mg/mL) and xylazine (1 mg/mL) in saline: 0.1 mL of this mixture was injected intraperitoneally per 10 g of body weight. The other control group of four guinea pigs was not challenged.

In the second study (vaccine B), four groups each of eight guinea pigs were injected subcutaneously which included: (1). 30 μg (OSP of vaccine B containing 2% Alhydrogel (InvivoGen, San Diego, CA, USA); (2). 3 μg of Q-VAX; (3). no vaccination and no challenge; and (4). no vaccination and challenged. Animals were challenged as per the first study, except challenge was 30 days post vaccination. 

In both studies, animals were only given one dose of the vaccine.

Seven days prior to challenge and for twenty-one days after, the guinea pigs were monitored daily for temperature, and three times a week for weight (Monday, Wednesday, Friday). A febrile response was defined as 40^o^ C or greater. Guinea pigs’ temperatures were analysed between days 4 and 13 post challenge, and weights were analysed at day 14 post challenge.

### 2.7. Microimmunfluoresence (IF) Serology of Guinea Pig Serum against C. burnetii

Glass slides coated with either phase I or phase II *C. burnetii* were reacted with a series of doubling dilutions of the guinea pig serum (1:25 to 1:3200). Slides were washed with phosphate buffered saline, then reacted with fluorescein-labelled goat anti-guinea pig IgG (H+L) secondary antibody (ThermoFisher, Waltham, MA, USA, Cat# A18884) at a dilution of 1:1000. Slides were then washed, dried, covered with mounting fluid, cover slipped and examined by UV microscopy. Each slide contained a negative and positive control serum. The highest guinea pig serum dilution that fluoresced similarly to the positive control serum was deemed to be the antibody titre of the serum.

Only guinea pigs vaccinated with vaccine A were tested, four animals were bled by cardiac puncture prior to challenge (day 58) and a further seven bleeds were taken after euthanasia, 22 days post challenge (day 80).

### 2.8. Quantitative PCR Assay for Detecting DNA of C. burnetii in Axenic Cultures and Guinea Pig Tissues 

A qPCR method was used for both measuring the concentration (genome equivalents) of *C. burnetii* in liquid axenic cultures and in infected mouse spleens and guinea pig tissues. Only guinea pigs vaccinated with vaccine B were tested for residual *C. burnetii* DNA in their tissues by qPCR. At 21 days post challenge, the guinea pigs were euthanised by a lethal dose of pentabarbitol. The spleen, a lobe of liver and one kidney were removed from each animal and placed in a pre-weighed sterile container, and the weight of each tissue sample was determined. One gram of each tissue type was homogenised using a hand homogeniser (Kimble, Rockwood, TN, USA, model # 749610-0015, size 15), in 2 mL of HBSS.

DNA extractions were performed using the DNeasy Blood and Tissue kit (Qiagen, Hilden, Germany). *C. burnetii* cultures were extracted following the manufacturer’s protocol for Gram-negative bacteria. 

For guinea pig tissue and mouse spleen homogenates, the samples were vortexed and 100 µL was added to a 1.5 mL screw cap tube with 100 µL sterile PBS. This was centrifuged at 10,000× *g* for 8 min and the supernatant removed. The tissue pellet was resuspended in 180 µL Buffer ATL to which 20 µL Proteinase K was added, followed by incubation overnight at 56 °C. The lysates were extracted following the manufacturer’s protocol for the purification of total DNA from animal tissues. The DNA was eluted in 100 µL Buffer AE (Qiagen) and stored at 4 °C until qPCR was undertaken. 

The extracted DNA was amplified using a qPCR assay with *C. burnetii*-specific Com1 primers and probe (forward 5′-AAAACCTCCGCGTTGTCTTCA-3′, reverse 5′-GCTAATGATACTTTGGCAGCGTATTG-3′, probe 5′- /FAM/AGAACTGCC/ZEN/CATTTTTGGCGGCCA/3IABkFQ/-3′) (Integrated DNA Technologies, IDT, USA). For guinea pig tissues, this was performed as a duplex assay, including primers and probes for the guinea pig housekeeping gene glyceraldehyde-3-phosphate dehydrogenase (GAPDH) (forward 5′-AATGGGAAGCTCACAGGTATGG-3′, reverse 5′-ATGTCATCGTATTTGGCCGGT-3′, probe 5′-/5Cy5/TCCAGGCGG/TAO/CAGGTCAGATCCACA/3IAbRQSp/-3′), to confirm the success of the DNA extraction. A standard curve was included to determine target copy number, comprising a serial tenfold dilution from 10,000 to 1 copy/reaction of a synthetic gene fragment (gBLOCK, IDT, USA) designed for each assay. The Com1 copy number was used to determine genome equivalents of *C. burnetii* based on this being a single-copy gene. qPCRs were performed on the MIC Realtime PCR platform. The qPCR reaction comprised 10 µL qPCRBIO Probe mix No Rox (PCR Biosystems, USA), 400 nM each primer, 200 nM probe, 5 µL template DNA, and nuclease-free water in a total volume of 20 µL. The cycling conditions were 50 °C for 2 min, 95 °C for 2 min, and fast cycling of 95 °C for 10 s and 60 °C for 20 s with fluorescence acquisition for 45 cycles.

GAPDH results for spleen tissue had Cq values of 21.9 ± 3.4 (mean ± SD), liver had Cq values of 27.0 ± 1.4 and the kidney had Cq values of 25.6 ± 2.8, indicating the DNA extractions were successful. The *C. burnetii* load was calculated as the copy number of Com1 genes per 100 mg wet weight of tissue.

### 2.9. Statistical Analysis

Vaccine A: The mean number of febrile days per guinea pig in each group was compared by analysis of variance, using Stata 15.1 software. Differences in weight at baseline and at 14 days post-challenge were compared using analysis of covariance.

Vaccine B: Temperature measurements (during the febrile period) and weight changes (14 days after challenge) were analysed using generalised linear mixed models. Organ data (presence/absence) for the four groups were analysed using a generalised linear model. All computations and analyses were performed in the R statistical software.

## 3. Results

### 3.1. Analysis of Purified OSP and OSP-TT Conjugates

The results of assays performed on purified OSPs derived from embryonated eggs and axenic media and their subsequent conjugates are presented in Table 1 and Table 2. Protein and nucleic acid contamination was lower in the OSP derived from axenic media compared to that grown in eggs. Contamination was at an acceptable level compared to other polysaccharide vaccines (i.e., <1.0% for protein and <2.0% for nucleic acid) for the axenic derived OSP, but above the acceptable level for the egg derived OSP, particularly nucleic acid. The size of the OSPs were assessed by size exclusion chromatography (results not shown), and both the egg and axenic media derived OSPs were estimated to be about 5000 Dalton.

The recovery of OSP (axenic media) and TT obtained after preparation of vaccine B was 83.4% and 100%, respectively. The recoveries obtained with vaccine A (egg derived OSP) were considerably less, with 30.7% and 46.3%, respectively. OSPs and vaccines A and B were prepared in different laboratories so the differences in recoveries could have been due to multiple factors. The w/w and molar ratios of OSP: TT were similar for vaccine A and B, with around 40 OSP saccharide chains per TT. 

### 3.2. Egg Derived OSP–TT Conjugate (Vaccine A) Protects against Virulent C. burnetii Challenge

The number of febrile days and weight changes following vaccination and challenge are presented in Table 3. Post challenge with virulent *C. burnetii,* guinea pigs vaccinated with OSP–TT conjugate prepared from OSP derived from egg grown *C. burnetii* (Vaccine A) developed significantly fewer febrile days than unvaccinated animals (*p* = 0.005). All unvaccinated animals became febrile for between 1 to 6 days with an average of 3.9 days: only three of the vaccinated animals became febrile, with an average of 1.0 day. The former became febrile approximately one day before the latter. The four negative control guinea pigs (not vaccinated, not challenged) did not become febrile during any day of the 10-day monitoring period.

The unvaccinated guinea pigs lost on average 13.6% of their body weight, whereas the vaccinated animals lost significantly less weight, 5.8% (*p* = 0.017). The control animals (unvaccinated, no challenge) gained 2.4% of their body weight. 

The results demonstrated that Vaccine A provides significant protection as determined by fever and weight loss after challenge with virulent *C. burnetii*.

Serology (Table 4) performed on vaccinated guinea pigs showed that four animals (three not tested) did not develop a detectable antibody against either phase I or II *C. burnetii* by day 58. Post challenge, six of the seven vaccinated animals were seropositive to both phase I and II *C. burnetii* at day 80 (22 days post challenge).

### 3.3. OSP from C. burnetii Grown in Axenic Media (Vaccine B) Equivalent to OSP Grown in Eggs (Vaccine A)

Fever and weight change results from the second study, where OSP–TT conjugate Vaccine B (OSP derived from *C. burnetii* grown in axenic media) was compared to Q-VAX, are presented in Table 5. Following challenge, two of the eight guinea pigs receiving Vaccine B developed fever, with an average of 0.4 febrile days: although, none of the animals receiving Q-VAX developed fever. All the animals in the unvaccinated group developed fever with an average of 3.9 febrile days. The former had their onset of fever approximately one day later than the latter. Vaccine B and Q-VAX demonstrated significant protection against fever (*p* = 0.005 and *p* = 0.001, respectively).

The unvaccinated guinea pigs lost on average 11.2% of body weight, and animals vaccinated with Vaccine B neither gained nor lost weight, demonstrating significant protection (*p* = 0.001) following challenge. Animals vaccinated with Q-VAX gained on average 3.2% of body weight post challenge. Q-VAX performed better than Vaccine B in terms of protection against weight loss; however, the difference between these groups was not significant (*p* = 0.059). Control animals (unvaccinated, no challenge) did not develop fever and gained 6.4% of body weight.

Results of *C. burnetii* DNA detected in guinea pig organs 3 weeks post challenge are presented in Table 5. *C. burnetii* DNA was detected in the spleens, livers and kidneys of six, six and eight (of eight) animals, respectively, that were unvaccinated and challenged with *C. burnetii,* indicating a systemic spread of the bacterium in unvaccinated animals. A large reduction in the amount of detectable DNA was seen in animals vaccinated with either Vaccine B or Q-VAX. The reduced amount of DNA detected in organs indicated that both vaccines the reduced systemic spread of *C. burnetii* within the guinea pigs. Unfortunately, low levels of Com1 DNA were detected in four organs (three kidneys and one liver) of the unvaccinated, non-challenged animals, indicating possible exposure due to cross contamination within the BSL-3 animal handling facility during clinical monitoring of the guinea pigs.

## 4. Discussion

Q fever remains a significant health problem which is often underestimated due to inaccurate diagnosis. Most people that become infected with *C. burnetii* develop mild to severe symptoms, which usually resolve in about 10 days [40]. However, between 10–20% of patients develop a “post Q fever fatigue syndrome” which can last for years and is extremely debilitating affecting both employment and lifestyle [41]. Furthermore, a small percentage of patients, up to 5%, develop a chronic, localised infection which damages the endocardium and heart valves and may lead to death due to cardiac failure. Clearly, prevention of Q fever is a worthy objective, especially in populations at high risk, and vaccination is likely to be the most effective method of prevention. 

Q fever is caused by *C. burnetii* a gamma-proteobacterium, with its closest phylogenetic relative being *Legionella* spp. [42] and with which serological cross-reactions can occur [43]. Inactivated whole cell vaccines made with phase I *C. burnetii* are effective in preventing Q fever; however, they are reactogenic and difficult to deliver due to mandatory pre-screening of patients prior to vaccination. Phase I *C. burnetii* cells induce a protective immune response whereas phase II cells do not induce a protective response, suggesting that the protective antigen is the OSP component of the phase I LPS. Polysaccharides tend to be poorly immunogenic, inducing a T-cell independent immune response, whereas protein conjugated polysaccharides induce a much stronger T-cell dependent response. The studies reported here were designed to determine if a phase I *C. burnetii* OSP conjugated to TT was protective.

The OSP in Vaccine A was prepared using existing methodologies (Section 2.2). However, these methods present problems for large scale manufacture and could raise problems with vaccine regulatory authorities. It was therefore decided that, for the second study, Vaccine B would be prepared with OSP purified from *C. burnetii* grown in axenic media rather than embryonated eggs, and that the purification of the OSP would be simplified. 

Vaccines A and B both protected guinea pigs from challenge with virulent *C. burnetii* indicating that OSP derived from culture in axenic media was equivalent to that derived from eggs. After the vaccine trial was performed, analysis using liquid chromatography mass spectrometry showed that the relative abundance of virenose and dihydrohydroxystreptose was comparable in egg and axenic media derived OSPs (results not shown). These two monosaccharides are thought to be critical for the induction of a protective immune response and are only present on phase I *C. burnetii*. The development of a protective immune response and the presence of the two monosaccharides indicate that one passage of phase I cells in either eggs or axenic media do not result in a phase change to phase II cells. The capacity to use axenic media to produce OSP is a huge advantage for vaccine manufacturing as it avoids the need to set up an expensive facility to cater for embryonated eggs under stringent Biosafety Level 3 (BSL3) conditions. The success of Vaccine B demonstrated that growth of *C. burnetii* in axenic media is a suitable platform for the production of OSP. Further work needs to be conducted to improve yield post purification (currently less than 10%) and to demonstrate a scale up of the process.

The reactogenicity of whole cell vaccines prepared from Gram-negative bacteria is primarily due to the lipid A component of the LPS. Preliminary studies in mice with Vaccine A did not reveal any reactogenicity, whereas mice receiving an equivalent dose of LPS were clearly unwell, with several mice dying. Furthermore, a small number of guinea pigs, that were immune after recovery from infection, did not show signs of reactogenicity after receiving a subsequent dose of Vaccine A. Future studies will need to demonstrate that multiple doses of the conjugate vaccine do not induce adverse reactions in previously exposed animals, of several species, before this vaccine could be considered for use in humans.

No detectable antibodies to *C. burnetii* (either phase I or II) were seen in guinea pigs receiving Vaccine A prior to challenge. Post challenge, a robust antibody response to phase I cells developed, most likely, a secondary anti-OSP response. Additionally, an antibody response to phase II cells developed; likely an anti-surface protein response given that phase II cells are devoid of full-length OSP. The response to phase II cells were previously shown to not protect against virulent *C. burnetii* but, nevertheless, there may well be an anti-protein antigen component of immunity playing a role in overall protection in Q fever. It was not possible to determine the mechanism of protection from the results presented here, but it is likely that anti-OSP antibody is involved, even though it was below the level of detection after one dose. Given that *C. burnetii* is an intracellular pathogen, it is probable that T-cell immunity/cell mediated immunity (CMI) plays a role in recovery from infection [44,45] and possibly hypersensitivity, also [46]. Whatever the mechanism of protection, clearly the OSP–TT conjugate vaccine is capable of priming the immune system to induce protection against virulent *C. burnetii* challenge.

In conclusion, this study showed that a single dose of conjugate vaccine consisting of OSP purified from phase I *C. burnetii* bound to TT protects guinea pigs from challenge with virulent *C. burnetii*. Additionally, it was demonstrated that OSP derived from *C. burnetii* cultured in axenic media is equivalent to that derived from egg grown cells. It will now be essential to test two or more doses of the vaccine to ensure that multiple exposures to the vaccine are not reactogenic, and to determine if multiple doses are more immunogenic and induce a higher level of protection than a single dose. Further studies will also examine dose responses to determine an optimal dose.

## 5. Patents

The results described in this paper are the subject of granted United States Patent No. 11,045,536.

## Figures and Tables

**Table 1:** Analysis of OSP derived from different sources.

Source of OSP	Derived from	Protein % mg/g of OSP	Nucleic Acid % mg/g of OSP	Endotoxin EU/mL
Slovak Academy of Sciences	Embryonated eggs	1.1	4.4	6.3 × 10^5^
Deakin University	Axenic media	0	1.9	Not Done

**Table 2:** Analysis of OSP-TT Conjugates.

	OSP Recovery %	TT Recovery %	Ratio of OSP: TT	
			*w*/*w*	Molar Ratio ^c^
Vaccine A ^a^	30.7	46.3	1.3	39
Vaccine B ^b^	83.4	109.0	1.5	45

^a^ OSP from Slovak Academy used to produce Vaccine A. ^b^ OSP from Deakin University used to produce Vaccine B. ^c^ Assuming MW of TT is 150,000 Dalton and OSP is 5000 Dalton.

**Table 3:** Protection induced by Vaccine A ^a^ measured by febrile response and weight change.

Group	1	2	3
Dose of vaccine (µg)	Nil	Nil	30
Challenge on day 57	No	Yes	Yes
Animals per group	4	8	7
No. of febrile days ^b^	0	3.9	1.0 ^d^
Average weight (g)	590	639	649
Weight change (%) ^c^	+2.4	−13.6	−5.8 ^e^

^a^ Vaccine A. OSP used to prepare OSP-TT conjugate obtained from *C. burnetii* grown in embryonated eggs. Conjugate dose: 30 μg intramuscular without adjuvant. ^b^ Febrile days (temp. ≥40 °C) measured from day 4 to 13 post virulent *C. burnetii* challenge. ^c^ Weight changes were measured 14 days post challenge. ^d,e^ Febrile response (*p* = 0.005) and weight loss (*p* = 0.017) significantly less in vaccinated group 3 compared to challenged control group 2.

**Table 4:** Serological response to Vaccine A measured by micro-immunofluorescence.

	Number of ANIMALS Tested ^a^	GMT (Immunofluorescence Serology)	
		Phase I Cells	Phase II Cells
Pre-challenge (Day 58)	4	<25	<25
Post challenge (Day 80)	7	>800	>1600

^a^ From the vaccinated group of seven, only four guinea pigs were bled by heart puncture one day prior to challenge (day 58), but all seven guinea pigs were bled post challenge on day 80. Phase I cells, the virulent form of *C. burnetii* have intact OSP present on their LPS. Phase II cells, the avirulent form are devoid of full-length OSP in their LPS.

**Table 5:** Protection induced by Vaccine B ^a^ measured by febrile response and weight change.

Vaccine Administered	None	None	Q-Vax	Vaccine B
Dose of vaccine (g)	Nil	Nil	3	30
Challenge on day 30	No	Yes	Yes	Yes
Animals per group	8	8	8	8
No. of febrile days	0	3.9	0	0.4
Average weight (g)	693	836	874	847
Weight change (%)	+6.4	−11.2	+3.2	0
DNA ^b^ detected in organs Number of animals positive (Geometric Mean of (positive only) samples)				
Spleen	0	6 (453)	0	1 (178)
Liver	1 (854) ^c^	6 (2167)	1 (1094)	2 (578)
Kidney	3 (462) ^c^	8 (5976)	2 (527)	3 (3092)

^a^ Vaccine B. OSP used to prepare OSP-TT conjugate obtained from *C. burnetii* grown in axenic media (ACCM-D). Conjugate dose: 30 μg subcutaneously with Alum adjuvant. Febrile days and weight changes were measured as per Vaccine A. Febrile response and weight loss significantly less in Vaccine B group compared to unvaccinated control group *p* = 0.005 and *p* < 0.001, respectively. ^b^ *C.burnetii* DNA in organs of guinea pigs: Copy number of Com1 gene per 100 mg wet weight of tissue, determined by qPCR. ^c^ *C.burnetii* DNA in the organs of non-challenged guinea pigs, presumably due to exposure and asymptomatic infection via the contaminated laboratory environment during clinical monitoring.
